# Reply to ‘Comments on two recent publications on GM maize and Roundup**’**

**DOI:** 10.1038/s41598-018-30751-9

**Published:** 2018-09-03

**Authors:** Michael N. Antoniou, Robin Mesnage, Sarah Agapito-Tenfen, Gilles-Eric Séralini

**Affiliations:** 1 Gene Expression and Therapy Group, King’s College London, Faculty of Life Sciences & Medicine, Department of Medical and Molecular Genetics, 8th Floor, Tower Wing, Guy’s Hospital, Great Maze Pond, London, SE1 9RT United Kingdom; 2 Genøk, Center for Biosafety, The Science Park, P.O. Box 6418, Tromsø, 9294 Norway; 3 CropScience Department, Federal University of Santa Catarina, Rod. Admar Gonzaga 1346, 88034-000 Florianópolis, Brazil; 4 University of Caen, Institute of Biology, EA 2608 and Network on Risks, Quality and Sustainable Environment, MRSH, Esplanade de la Paix, University of Caen, Caen, 14032 Cedex France

## Abstract

The opinion expressed by Eriksson and colleagues’ fails to recognise that there are no standard experimental designs for academic investigations involving omics analyses of genetically modified crops and that the only valid comparator to determine the effect of the process of transgenesis is a near isogenic variety grown at the same time and location, as was the case in our investigation of NK603 maize. Eriksson does not acknowledge that the quality of the rat liver tissues in our chronic Roundup toxicity study has neither been questioned nor branded as unsuitable for further investigation. In addition, Eriksson fails to appreciate that the statistical methods we used to analyse the liver metabolomics dataset are recognised as appropriate as some of a number of approaches that can be taken. Moreover, Eriksson neglects to mention that the proteomics analysis of the liver tissues highlights structural and functional damage from Roundup exposure. Thus our results are sound and the claims by Eriksson and colleagues of experimental flaws are unfounded.Replying to: Eriksson *et al*. Sci Rep 8 (2018); 10.1038/s41598-018-30440-7.

## Introduction

### NK603 study^1^

Eriksson criticizes us^2^ for not including spatially separated biological replicates and for analysing only 2 temporal replicates. However, our study aims were strictly restricted to identifying potential metabolic differences between NK603 genetically modified (GM) maize and a near-isogenic control grown under agricultural conditions. While some requirements for GM crop field trials performed by industry for regulatory purposes are set down in EU law, there are no standard experimental designs for academic investigations involving omics analyses; some use the suggested randomised block field design^3,4^, most do not^5–7^. Moreover, we assessed the consequences of Roundup herbicide application, which has not previously been undertaken^3^. Most GM vs non-GM omics investigations use one environmental and temporal replicate to test equivalence^4,8–13^. We acknowledged further experiments are needed under different environmental conditions to determine the full range of GM process effects on this maize type. However, this does not invalidate our results.

Eriksson complains we did not include evidence to show that the non-GM control variety is near-isogenic to NK603. GM material available in the marketplace is always backcrossed with other varieties and the pure isogenic is never available for independent research. Furthermore, no international guidance defines ‘near-isogenic’. Therefore this is a judgment call. We used a type of NK603 (Monsanto/Dekalb DKC 26–78) and the nearest available isogenic line (Monsanto/Dekalb DKC 26–75). The use of other near isogenic non-GM lines, if available, would constitute additional valid comparators, and provide useful information about variability between related strains. However, for GM vs non-GM comparative studies conducted both by those based in academia as well as those in industrial settings for regulatory purposes, the usual method is to employ a single near-isogenic non-GM comparator. For example, in the study of NK603 GM maize conducted by the developer company (Monsanto), only a single near-isogenic non-GM comparator was used, although no information as to its exact nature was provided^14^. Thus our experimental design of using only one near isogenic non-GM comparator is the norm within the field and the degree of detail we provide of the non-GM near isogenic maize variety used^1^ exceeds that disclosed by industry^14^.

Eriksson states that there is a lack of reference to expected natural variation in the levels of proteins and metabolites among different maize cultivars in our investigation. However, the point of our study was to assess the effect of the GM process on the composition of NK603. Thus the only scientifically valid comparator is the non-GM closest (isogenic) relative. Comparisons with different maize varieties that do not constitute near-isogenic strains, and which are grown at different locations and times, would only serve to increase variation in the dataset and thus mask rather than highlight the effects of the GM process, negating the purpose of the study. Additional valid comparisons could be made between other maize strains harbouring the NK603 event (produced by out-crossing of the original genetically engineered parent) and their near-isogenic maize variety(s), always with the caveat that they must be grown at the same time and location. Such an investigation would determine if our findings are unique to the NK603 strain we have studied or occur generally with this genetic modification event.

We agree that the cadaverine and putrescine content of maize varies; this is covered in our Discussion.

### Roundup study

Rat liver tissues were obtained^15^ from animals described in a previous report^16^. Eriksson tries to discredit the findings of our study by association with this previous investigation^16^, which sparked much controversy^17–23^. Livers were freshly excised from euthanized animals, snap frozen and appropriately stored to maintain integrity. No evidence exists suggesting that these tissues are unsuitable for experimental use. The influence of age and the presence of tumours was not a concern. This can be independently verified as the raw data (age, tumour presence) are available^15,16,24^.

We did not overlook the consequences of the metabolomics Benjamini-Hochberg false discovery rate. We provide careful interpretation and highlight limitations in the Discussion (Lines 5–12, page 6; Lines 17–26, page 9). Numerous statistical methods have been applied to extract biologically meaningful information from metabolomics^3,5,6^. We employed methods that are recognised as appropriate by experts in the field. Surprisingly, Eriksson neglects to acknowledge our proteomics analysis, which provides data of high statistical significance revealing damage from Roundup ingestion. Therefore our bioinformatics and statistical analyses of both proteomics and metabolomics are sound and when taken together provide a consistent pattern of liver structural and functional defects.

Thus the claims by Eriksson and colleagues of experimental flaws in our investigation of both NK603 maize composition and chronic ultra-low dose toxicity of Roundup are unfounded.
